# Topics of Public Interest

**Published:** 1915-01

**Authors:** 


### TOPICS OF PUBLIC INTEREST.

---

**Belgian War Losses.** According to Figaro of Paris, the Belgian losses, since the beginning of the war have been about 25,000 killed; 52,000 wounded; 35,000 captured, and 32,000 interned in Holland. It is interesting from the medical standpoint to note the popular error with regard to the direct mortality of warfare. The idea that a country can be depopulated by war on a large scale and under modern conditions is true to a small degree and in a somewhat indirect way. Anyone who holds this idea and who studies the statistics finds the direct mortality almost disappointingly small, although for humanitarian reasons, he will hesitate to use such an expression. The full military strength of any average population is about a quarter of its total numeric strength; that is to say, half will be males and of these, half will be included between the ages of 20 and 55. With allowance for disabled men and those required as non-combatants, it is only under stress that the military strength will reach 25% of the total population. At 15 per 1,000 mortality per annum, the average deaths in four months for Belgium, whose population is about 7,500,000 would be nearly 37,500. The direct mortality from warfare is about twice the peace mortality for the entire male, adult population. This is, of course, a serious increment but probably not so important numerically as the increase of mortality among noncombatants, from exposure, fatigue, excitement, insufficient nourishment, etc. Belgium has been depopulated temporarily, to a much greater extent than by direct mortality of war, since it is estimated that about 2,000,000 have taken refuge in France and about 1,000,000 in Holland and England. Even if the war were now over, the mortality of non-combatants would continue at a high rate for a year or two more.

---

**The Cartwright Biennial Prize,** established by the alumni of the College of Physicians and Surgeons, is open to all, with free choice of subject. Essays must be sent before April 1 to Dr. H. E. Hale, 770 West End Avenue, New York City.

---

**County and Auxiliary Lay Societies in Connection with State Sanitary Officers' Association.** This plan has been formulated by the President of the latter body, Dr. Montgomery E. Leary, of Rochester, and presented in an address published in "Health News," the Bulletin of the State Board of Health.

---

**License to Practice Special Branch of Medicine or Surgery.** California, Statutes of 1911, page 1437, provides for the issuing of a certificate by the Board of Medical Examiners, the qualifications being 35 years of practice, 15 within the states, in the specialty, and a practical examination. We do not understand that this law prohibits special practice without such license—indeed the time requirement could not reasonably be made on such a basis—but that the intent of the law is to enable advertising specialists who have not been regularly licensed, to acquire a legal basis or to provide a sort of endorsement for specialists of long practice. It is obvious that the conferring of such a license would imply a minimum age of nearly 60.

---

**The Charity Ball for the Benefit of the German Hospital,** will be held January 4, 1915.

---

**A Resolution in Favor of Woman Suffrage** has been voted down by the New York State Nurses' Association. We do not understand that this indicates a formal expression of opinion on the merits of suffrage but rather that any organization should stick to its own line of activity.

---

**The Founder of Cremation in America,** Dr. Francis Julius Lemoyne, was honored by the unveiling of a tablet at the commencement exercises of his alma mater, Washington and Jefferson College of Washington, Pa., at the last commencement.

---

**County Tuberculosis Hospitals** have been voted for Chenango, Lewis and Suffolk Counties, bringing the total to 29 counties in New York State, nearly 50%.

---

**Electrocution for Pennsylvania,** will begin with 1915, the last execution of the death penalty by hanging having recently occurred.

---

**Automatic Telephone Service** has been inaugurated in Rochester by the Federal Co. Intermural connections between Buffalo and Rochester will henceforth be made by the long distance operator, who will use the automatic dial for the opposite city, thus reducing delays. The automatic service has proved to be time saving and it eliminates all doubt as to accuracy except on the part of the caller. There are practically no blanks. Barring a defect of the calling line one always gets some number or else a special operator who gives notice of change of number or is automatically notified of a defect on the line called.

---

**The DeGraff Memorial Hospital** was formally presented on December 1, for the joint use of the cities of Tonawanda and North Tonawanda, title being held by the latter. The first floor contains four wards, emergency, operating room, offices, etc; the second floor contains 15 private rooms. 30 beds are already provided. Credit for the undertaking is largely due to Mayor John A. Rafter, M. D., formerly Associate Editor of this Journal.

---

**Niagara Falls Coroner's Report.** Dr. Walter A. Scott reported for the year ending November 1914, 133 cases, including 28 accidents and 8 suicides or attempted suicides. Ten fatal accidents were due to railroads.

---

**A Smokeless and Hot Flame,** for boiling a test tube, etc., can be obtained by igniting a tablet of Hexamethylene tetramine.

